# Geometry Shapes Evolution of Early Multicellularity

**DOI:** 10.1371/journal.pcbi.1003803

**Published:** 2014-09-18

**Authors:** Eric Libby, William Ratcliff, Michael Travisano, Ben Kerr

**Affiliations:** 1 Santa Fe Institute, Santa Fe, New Mexico, United States of America; 2 School of Biology, Georgia Institute of Technology, Atlanta, Georgia, United States of America; 3 Department of Ecology, Evolution, and Behavior, University of Minnesota, St. Paul, Minnesota, United States of America; 4 Department of Biology and BEACON Center, University of Washington, Seattle, Washington, United States of America; ETH, Switzerland

## Abstract

Organisms have increased in complexity through a series of major evolutionary transitions, in which formerly autonomous entities become parts of a novel higher-level entity. One intriguing feature of the higher-level entity after some major transitions is a division of reproductive labor among its lower-level units in which reproduction is the sole responsibility of a subset of units. Although it can have clear benefits once established, it is unknown how such reproductive division of labor originates. We consider a recent evolution experiment on the yeast *Saccharomyces cerevisiae* as a unique platform to address the issue of reproductive differentiation during an evolutionary transition in individuality. In the experiment, independent yeast lineages evolved a multicellular “snowflake-like” cluster formed in response to gravity selection. Shortly after the evolution of clusters, the yeast evolved higher rates of cell death. While cell death enables clusters to split apart and form new groups, it also reduces their performance in the face of gravity selection. To understand the selective value of increased cell death, we create a mathematical model of the cellular arrangement within snowflake yeast clusters. The model reveals that the mechanism of cell death and the geometry of the snowflake interact in complex, evolutionarily important ways. We find that the organization of snowflake yeast imposes powerful limitations on the available space for new cell growth. By dying more frequently, cells in clusters avoid encountering space limitations, and, paradoxically, reach higher numbers. In addition, selection for particular group sizes can explain the increased rate of apoptosis both in terms of total cell number and total numbers of collectives. Thus, by considering the geometry of a primitive multicellular organism we can gain insight into the initial emergence of reproductive division of labor during an evolutionary transition in individuality.

## Author Summary

A major transition in evolution occurs when previously autonomous entities become co-dependent in the context of a higher-level entity. Such transitions include the evolution of multicellular organisms from unicellular ancestors and eusocial “superorganisms” from multicellular ancestors. The evolution of reproductive division of labor occurs after some of these transitions (e.g., germ-soma differentiation in multicellular organisms). Yet, how exactly this occurs is unknown. Here, we examine this issue in the context of an experimental model of primitive multicellularity that evolved a form of reproductive division of labor *de novo*. Cells within groups evolved higher rates of death. Through cellular death, groups of cells split apart and formed new groups in a form of collective reproduction. The evolution of this trait is puzzling since cells originally formed groups under selection for large size. Group splitting produces smaller groups which are less likely to survive in the experiment. We show that the organization of the group is key to understanding evolution of increased cell death. Due to the arrangement of cells, higher rates of cell death increase both the number of cells and groups that survive. Reproductive division of labor evolves because the group context changes the fitness value of traits.

## Introduction

Organisms have increased in complexity through a series of major evolutionary transitions, in which formerly autonomous entities become parts of a novel higher-level entity [1]–[5]. Examples of this transition include the evolution of multicellular organisms from unicellular ancestors and eusocial “superorganisms” from multicellular ancestors. One of the primary benefits ascribed to major evolutionary transitions is the potential for the higher-level entity to evolve division of labor among its lower-level units [1], [2], a subject which has received a good deal of theoretical attention [6]–[11]. A salient form of this is reproductive division of labor in which some lower-level units forgo their contribution to reproduction of the higher-level entity. This is found in the germ-soma differentiation in multicellular organisms and worker/queen roles in eusocial insects [12]–[17]. While reproductive specialization is not strictly required for division of labor to provide a fitness benefit to the higher-level entity, it has evolved repeatedly in independent lineages [18]–[22].

Upon a superficial glance, the existence of such reproductive self-sacrifice seems to present an evolutionary paradox. How would such a self-destructive tendency be favored by a process (natural selection) that places a high premium on survival and reproduction? The resolution of the paradox generally involves a situation in which the self-sacrifice improves the fitness of the higher-level unit [6], [7], [9], [11]–[13], [17], [23]–[27]. For ease of discussion, let us call the lower-level entities “particles” and the higher-level entities “collectives”. Suppose the altruistic action of some particles allows other particles in their collective to found new collectives at a higher rate. If these founding particles possess a tendency for self-sacrifice (which can occur if particles have high relatedness within collectives), then reproductive division of labor within collectives can evolve. We emphasize that such altruism must occur in a strict subset of particles within the collective and requires the plastic or stochastic expression of phenotypic traits at the particle level. Thus, while the logic for the evolution of reproductive self-sacrifice is sound, the mechanistic underpinnings could be complex. The precise way in which such differentiation evolves and its presence in the early stages of major transitions are largely unknown.

A recent evolution experiment on the yeast *Saccharomyces cerevisiae* has provided a unique platform to address the issue of reproductive differentiation during an evolutionary transition in individuality [28]. In this experiment, populations of unicellular yeast were periodically exposed to a selective regime that rewarded cells that sank quickly in test tubes. During this setting, cells in clusters sink more quickly than independent cells, incentivizing group formation. Cluster-forming phenotypes evolved repeatedly via the retention of cell-cell connections after mitotic reproduction. These group-forming types outcompeted their unicellular ancestors, driving them to extinction in all 10 replicate populations within 60 days [28]. Clusters grew in size until the resulting physical strain caused them to fragment, yielding a form of group reproduction. As a result, the yeast evolved group formation and reproduction *de novo*. Interestingly, these yeast clusters soon evolved a secondary trait: a higher rate of cellular programmed death (hereafter referred to as apoptosis). Why would a higher rate of cellular suicide, an ostensibly costly trait, be favored by natural selection?

A higher rate of apoptosis might have evolved because it increases collective-level reproduction. Since each cell in the group is connected solely to its parent and offspring cells [28], it only takes a single break in any connection to produce two distinct collectives. Both physical strain and cell death can create such breaks and, consequently, increase the number of groups. Selection for a greater number of clusters could promote division of labor [6]–[11], providing an explanation for the evolution of higher rates of apoptosis. Yet, the problem is that the selective regime seemingly rewards large clusters (group size), not large *numbers* of clusters (group fecundity). Moreover, the apoptotic mechanism of group reproduction acts in direct opposition to group viability. While there may be a benefit for groups to reproduce (to reduce the risk of not being transferred due to random sampling error), as groups divide they become smaller and sink less quickly, making them less competitive against larger groups. It would appear that an optimal strategy would be for groups to grow as large as possible and divide infrequently. In contrast, when selection for large groups is stronger (requiring faster settling), groups evolve higher rates of apoptosis and produce proportionally smaller propagules [28].

To address this conundrum, we build a series of mathematical and computational models. The first model explores the optimal way for clusters to split under a selective regime similar to the Ratcliff *et al*. experiment [28]. We see that it is possible for high rates of cluster division to be adaptive, but the opposite is also a possibility. This first model ignores various details about the yeast system for tractability, including the geometry of the clusters and cellular apoptosis. The second model explicitly considers the cellular arrangement within yeast clusters and the consequences of apoptosis on cluster reproduction. This model reveals how the geometric structure of the cluster interacts with apoptosis to affect the number and size of cluster offspring. We find that the organization of snowflake yeast imposes powerful limitations on the available space for new cell growth. By dying more frequently, cells in clusters circumvent space limitations, and, paradoxically, reach higher numbers. Finally, we demonstrate that selection for particular cluster sizes can explain the increased rate of apoptosis both in terms of total cell number and total numbers of collectives. Thus, considering the specific geometry of the clusters reveals the adaptive benefit of the evolution of reproductive self-sacrifice in the Ratcliff *et al*. experiment [28], and a possible mechanism for the emergence of reproductive division of labor during an evolutionary transition in individuality.

## Results

### Optimal cluster division

In this section, we build an abstract model to get a rough understanding of how the experimental regime might select for different rates and forms of cluster division. When a cluster splits, it yields both new and smaller clusters. Thus, division simultaneously affects cluster reproduction and the prospects for viability under settling selection. How should a cluster balance fecundity against survival? Similar to the experimental regime of Ratcliff *et al*. [28], we use a framework with a growth phase followed by a selection event, enabling cluster division strategies to depend on time within the growth phase. Since cluster growth and division change the size of clusters, we permit splitting strategies to be size-dependent. Here we use a dynamic programming approach [29], [30] to explore optimal cluster division strategies.

We denote the probability a cluster of 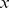 cells survives settling selection as 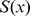. Since larger clusters settle faster than smaller ones, we assume 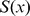 is a non-decreasing function. In addition, we assume that division and growth of clusters occur for 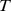 time steps prior to settling selection. We define 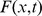 to be the maximal reproductive output for a cluster of size 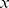 (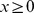) at time 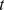 (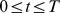). As a consequence, if fitness is measured in terms of number of clusters 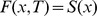, and if fitness is measured in terms of the number of cells 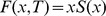. Over each time step (from 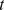 to 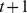, where 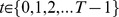), we assume that clusters divide and then grow. Specifically, a cluster of 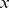 cells at time point 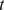 splits into two clusters of sizes 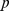 and 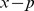 (where 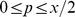). We note that this includes the case where the cluster does not divide (i.e., 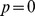); therefore, this framework allows us to track optimal cluster division *rate* as well as optimal size for cluster propagules. After division, the new clusters grow according to the function 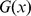; that is, a cluster that starts with 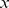 cells ends the time step with 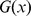 cells. For instance, if every cell in a cluster doubles over a time step, then 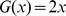. We have the following backwards recursion for maximal reproductive output:
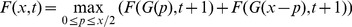
(1)


Suppose fitness is measured in terms of the number of clusters that survive selection (i.e., 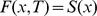). In the Supplement, we prove that if 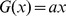, where 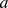 is some positive integer greater than 1 and 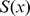 is concave (e.g., 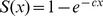), then the optimal strategy is always to divide into halves (or as close to halves as possible). If cell death is the means of cluster division, these conditions would predict the evolution of cell death mechanisms that produce equal sized cluster offspring. Generally in this case, higher splitting rates could be favored and cell death may be one way to accomplish this.

However, there are several important caveats regarding this result. If 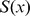 is convex over some range of 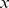 as might be found in a Hill function, then it can be optimal not to divide at all (at least for some sizes; see Supplement). If fitness is measured in terms of the number of cells (i.e., 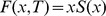) rather than the number of clusters, it can be optimal not to divide even when 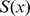 is strictly concave (see Supplement). In such cases, cell death rate would be predicted to decrease.

Furthermore, the model lacks a mechanistic basis for cluster division. Such a basis follows from recognizing the geometry of the cluster. Yeast clusters form when mother cells remain attached to their budding daughter cells. Because a given mother cell can have multiple attached daughters, the cluster is a branched acyclic network (i.e., a multi-branched tree). Suppose cell death severs a single cell-cell connection. In such a case, a yeast cluster will produce two daughter clusters. However, the sizes of the daughter clusters are constrained by the network topology of the mother cluster. In terms of the above model, some 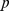 values will not be possible and breaking a random link will make some 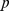 values much more likely than others. Consequently, the model's implicit assumption that any value of 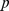 is equally available to a dividing cluster is misplaced. Moreover, if the rate of cell death is constant, then larger clusters should expect more broken links (and therefore more offspring clusters per unit of time), which is not currently captured by the above model. To address these issues directly, we explicitly incorporate cluster geometry into our second model.

### Cluster structure, growth and reproduction

**Figure 1:**
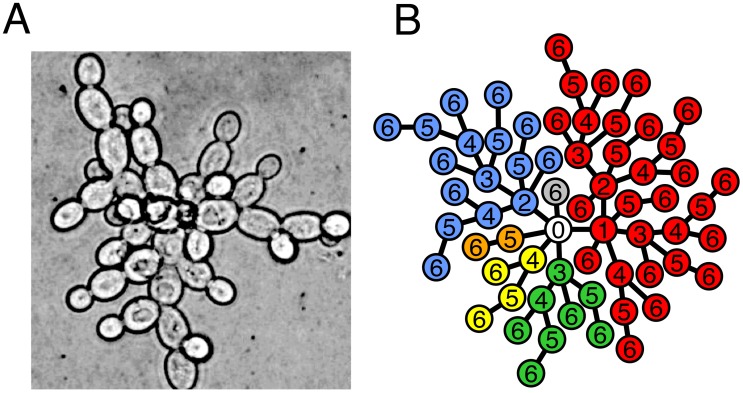
Groups as trees. **A**) Photograph of a cross-section of the yeast snowflake phenotype shows the branching morphology. **B**) Simulated group growth from a single cell (*Node 0*) after 6 rounds of cell reproduction (generations). The different colors represent different branches emanating from *Node 0*. The numbers inside nodes represent the generation of their birth.

We describe the structure of a cluster by a tree graph in which nodes represent cells and edges represent physical attachments between cells (Figure 1). When a cell reproduces, its corresponding node in the tree gains an edge to a newly created node. This growth mechanism ensures that cells are only attached to their parent and offspring. For simplicity we begin the tree with only one node which represents the first mutant yeast cell to have the capacity to form clusters, call it *Node 0*. Each time *Node 0* reproduces it generates a branch which will continue to grow independently. Initially, we assume that all cells reproduce and do so at the same time. So with each successive generation the tree doubles its nodes, i.e. the cluster doubles in the number of cells. After 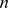 generations the branches from *Node 0* will be composed of 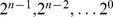 cells depending on when the branch was initiated. The total number of cells in the tree is 
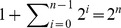
 (we note this is equivalent to 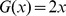 in the model from the previous section).

Due to the tree geometry, if a link/edge between two cells/nodes is severed then it will result in two distinct clusters, i.e. the cluster reproduces. Since both physical strain and cell death lead to cluster reproduction, we can view these as mechanisms for severing an edge between two nodes. It is also possible to divide clusters by removing a node rather than severing an edge. Removing a node with more than two connections, however, could result in “multiple births”, which is not typically observed experimentally. Thus, we assume clusters reproduce via link severance. Furthermore, in order to explain the experimental observations of Ratcliff *et al*. [28], we consider cell death as the primary mechanism of link severance–although other mechanisms may also exist. Whatever the mechanism, the location of the severed edge plays a significant role in determining the sizes of the resulting cluster offspring. If an edge in the periphery is severed then one of the resulting clusters will be composed of only a single cell. In contrast, severing more central edges will result in more symmetry between offspring clusters.

The particular manner by which cells die determines whether a severed edge is more likely to be in the periphery or the center. If cell death is completely random such that the centermost cells are just as likely to die as newly created cells and the tree is doubling in size every generation, then the severed edge is more likely to be peripheral. This is because at any time 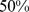 of the tree is newly created. As a result, there is a 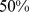 chance that death of a random cell will yield a “group” that is one (dead) cell in size by breaking its one and only link to the tree. The expected sizes of the offspring clusters after 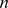 rounds of cell reproduction are 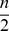 and 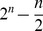, and the ratio of the smaller offspring to the parent is less than 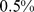 after 10 generations (the ratio, 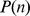, after 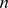 generations is 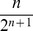). Such a small cluster may not be able to grow large enough to survive the selective regime and could be excluded from future growth and reproduction.

If cell death is not completely random but rather related to age then central edges would be more likely to be severed. In the case that the oldest cell (*Node 0*) dies, the sizes of the resulting offspring clusters will depend on which link is severed. Each link of *Node 0* corresponds to one of its branches with 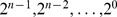 cells. Without a bias as to which link is severed the smaller offspring would be expected to have 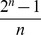 cells. The ratio of this offspring to the parent after 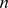 rounds of cell reproduction, 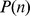, is 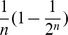. After 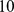 generations 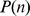 is approximately 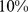 which is 20 times larger than when cell death is completely random. Thus, weighting death towards older, more central cells increases the size of the smaller offspring.

Experimental observations of early cluster offspring in the yeast system suggest that the smaller offspring may be closer to 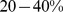 of the size of the parent [28]. To see how link severance via cell death can achieve such values, we consider again the death of *Node 0* which yielded less offspring asymmetry than random cell death. The oldest branch of *Node 0*, created in the first round of cell reproduction, is the only branch greater than 40% of the tree size– it is half of the size of the whole tree. The next oldest branch, created the second time *Node 0* reproduces, is a fourth of the whole tree size. Each successive branch is half the size of the previous. If there is no bias in which branch becomes the offspring then the odds favor the 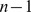 branches that are much less than 40%. Instead of unbiased link severing, it could be that links are severed according to the size of the branch they are supporting. Bigger branches may produce more strain on their links compared to smaller branches and, therefore, may break more easily. Although there are many potential ways to bias severance in favor of bigger branches, we assume a simple biasing such that the probability a link is severed is directly proportional to the size of the branch. In this case, the ratio of the smaller offspring to the parent after 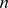 generations, 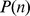, is 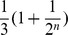 which approaches 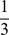 as 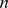 increases. This matches experimental observations more closely and suggests that cluster division via cell death may be biased both in which cells die and which links are severed.

### Growth constraints

**Figure 2:**
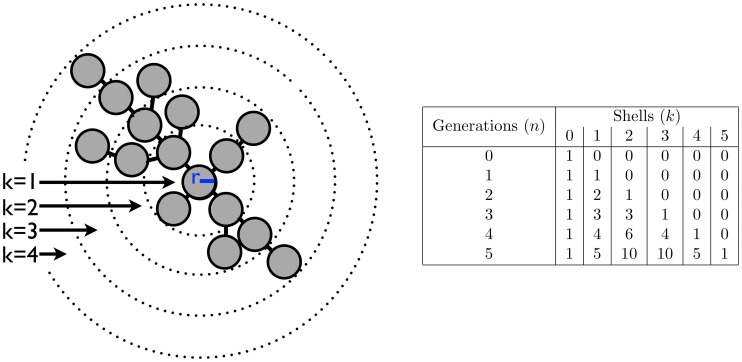
Volume constraints to tree growth. (**Left**) A model of the growing tree with *Node 0* at the center and shells of nodes surrounding it. Each cell is a sphere with radius r and the edges are only shown to make relationships clear– edge length is effectively 0. (**Right**) After 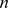 generations, each shell 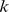 contains 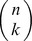 cells exclusively.

Until now, we have operated under the unrealistic assumption that all cells in a cluster have the same constant rate of reproduction. Although each time a cell reproduces, the cluster increases in size and span; it also fills the limited volume at the center. As this space gets crowded, cells lose both access to nutrients and room for further reproduction. To determine how the tree geometry experiences volume constraints, we use a 3-dimensional model of growth in which cells occupy concentric shells surrounding the central node, *Node 0* (Figure 2). By stretching the cluster along its longest diameter, this model maximizes the available space and sets an upper bound to the size capacity of the cluster.

We assume that each cell is an identical sphere with radius 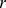. Cells occupy shells depending on how many links separate them from *Node 0*. For example, the third shell is filled with cells that are 3 links from the center. The offspring of a cell occupies the next shell and, conversely, its parent is in the previous shell. Each shell 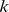 encloses a volume equivalent to a sphere with radius 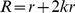. This volume (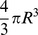) can hold at most 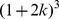 cells– ignoring issues concerning the maximum packing of spheres. In the growing cluster, the actual number of cells within the volume of a shell is simply the total number of cells in each interior shell. For a given shell 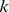 after 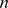 rounds of cell reproduction the total number of cells is 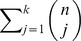 (see Figure 2). Thus, the volume enclosed by shell 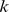 is exceeded when the number of generations 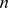 satisfies:
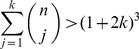
(2)


We calculate the lowest 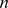 for which the volume bounded by each shell is exceeded and find shells 4–6 are the first to overflow at the twelfth round of cell reproduction (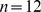). Even if a cell in shell 4 could relocate to shell 3 there is no room available because the volume defined by shell 4 has been exceeded. While there is still space in the volume contained by shells 7–12, cells from the overcrowded volume cannot move here because they must remain connected to their parents in more interior shells.

**Figure 3:**
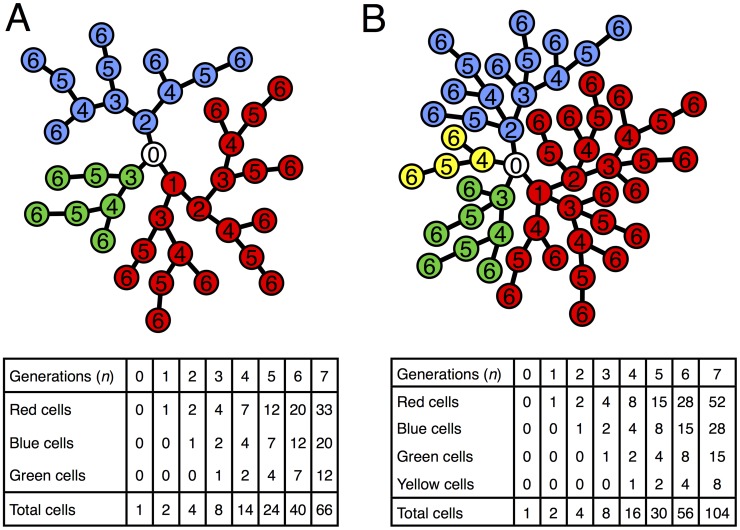
Degree capped tree growth. **A**) A model of a growing tree with *Node 0* at the center and a degree cap of 3. The numbers inside each node represent the generation of their birth while the colors denote the 3 different branches emanating from *Node 0*. The table below shows the total number of cells in each branch and the tree as a whole as a function of the number of generations. The number of nodes of each branch follow the same series: 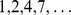 described by the recursion 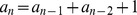. This can be solved analytically to get 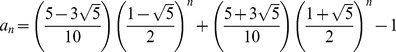. **B**) A model of a growing tree with a degree cap of 4. Similar to A) there is a recursive relationship for the number of nodes in a branch but it delves one more generation into the past, i.e. 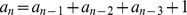. For both trees the total number of nodes in the tree is twice the number in the red branch.

In addition to the volume constraint, there may be constraints regarding how many attachments (edges) a single cell can have. Experimental observations of cluster structure find that most cells are attached to only a few cells (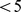). If there is a limit to the number of attachments per node then this will alter the organization of a cluster (Figure 3). For example, a tree with maximum node degree of 3 will have just 3 branches emanating from *Node 0*. Instead of doubling with each round of cell reproduction, the number of nodes in a branch follows a recursion: 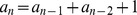, where 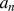 is the number of nodes 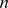 episodes after the creation of the branch. Geometries with higher maximum node degrees (hereafter called “degree capped”) also feature recursive relationships such that in general, for a tree with degree cap of 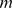, 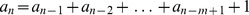 with the first 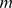 values following 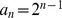. This stems from an important distinction in trees with a degree cap: their size only increases with those cells created within the last 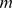 generations. These recursive relationships relate cluster sizes with Fibonacci numbers such that trees without degree caps are simply Fibonacci sequences of infinite order. In all cases, the total number of nodes in the tree is simply twice the number in the largest branch.

As the distribution of cells in branches is altered by limiting the number of node attachments so, too, is the expected size of offspring clusters. If *Node 0* dies and there is no bias to which link is severed then the expected offspring size as a proportion of the parent is 
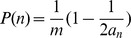
, where 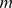 is the degree limit and 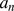 is the number in the largest branch. This quickly approaches 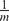 which is much greater than the value 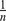 found in trees without limits to the number of node attachments. Consequently, cluster offspring are more equal in size. In trees without degree caps, biasing which link is severed according to branch size increased the symmetry of cluster offspring such that the expected size of the smaller offspring is 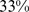 of the parent's size. By comparison, biasing link severance in favor of bigger branches (as done before) has less of an effect in trees with degree caps. The expected size of the smaller offspring is 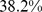 of the parent's size for a degree cap of 3 and 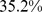 for a degree cap of 4. While the most symmetric cluster reproduction is in trees with a degree cap of 3 and biased link severance, all trees with biased link severance produce an offspring that is between 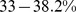 of the parent's size. It should be noted that a degree cap of 2 can do better but it can only form filaments rather than snowflake-shaped clusters.

Not only do trees with degree caps produce more symmetric offspring but they can experience less severe volume constraints. Since limiting the number of attachments per cell reduces the size of a cluster, it effectively delays when clusters begin to run out of space. A tree without degree caps can only go through 11 rounds of cell reproduction before exceeding the available volume contained by a shell. A tree with degree cap of 4, however, goes through 14 generations before encountering a limit at shells 8–11. A tree with a degree cap of 3 can undergo even more generations, exceeding shells 14 and 15 on the 20th round of cell division. It reaches 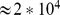 cells before encountering volume limitations which is twice that of trees with a degree cap of 4 (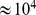 cells) and 5–10 times as many cells as trees without degree caps. Interestingly, trees with degree caps of 2 produce filaments, a common biological shape, that are free of any volume constraints.

### Population simulations

Thus far, we have examined the consequences on cluster reproduction of link severance due to death of a single cell. In practice, however, as clusters grow and reproduce, mechanisms of cell death interact with geometric constraints to create a population of clusters with a distribution of sizes. In addition, the death of a cell has downstream consequences by preventing future growth of a branch. To determine how such mechanisms interact, we simulate the population expansion from the first mutant capable of forming clusters, *Node 0* (see Methods).

**Figure 4:**
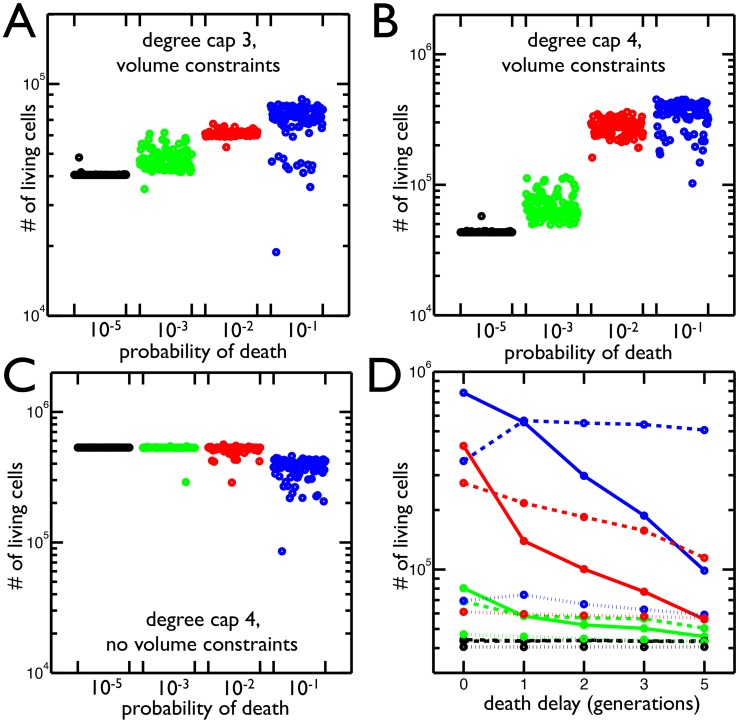
Total number of cells resulting from different rates of cell death and geometric constraints. **A**) Total number of living cells in groups with maximum node degree 3 in 100 simulations at 4 different probabilities of cell death. The highest probability of death (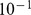 in blue) has the largest number of cells and the greatest variance in final cell number. **B**) Same as A but with maximum node degree 4. Once again the highest probability of death (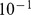 in blue) produces the most cells. **C**) The same as B but the volume constraints are removed, i.e. the maximum node degree is still 4 but there is no limit to the number of cells in each shell. The 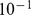 probability of death no longer increases the population of cells. **D**) The mean number of living cells when a cell's susceptibility to death is delayed by 0–5 generations (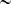 time units) since it last reproduced. The colors correspond to probabilities of death: 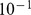 (blue), 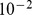 (red), 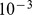 (green), 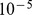
(black); and the line style represents the degree cap: no cap (solid), 4 (dashed), 3 (dotted). In trees with degree caps of 3 and 4, the highest probability of death results in even more cells when death is delayed one generation but less as death is delayed further. In all other cases, delaying death results in less cells.

The population simulations show that the total number of living cells increases with the probability of cell death (Figure 4A & B). This paradoxical result is a consequence of the constraints on cell reproduction due to degree caps and limited volume. For a degree cap of 3, cells that reach the maximum degree (3 in this case) stop reproducing. After 21 generations, many cells have reached the maximum degree and no longer contribute to the growth of the population. By dying, a link connecting two non-reproducing cells is broken. This allows one cell to reproduce again and start a new branch that increases the population by more cells than the cost of the dead cell. Since a cluster with degree cap of 3 does not encounter volume limitations until the 20th generation, near the end of the simulation, the volume constraint does not play a significant role in the increased cell population. In fact, it can be removed and the total number of cells still increases with higher rates of cell death. This is not true with clusters that have a degree cap of 4 (Figure 4C). The higher degree cap reduces the extent to which fixing a maximum number of attachments constrains the population while at the same time increases the strength of the volume constraints– cells experience volume limitations by the 14th round of cell reproduction. So, both degree cap and volume constraints allow clusters to increase the number of living cells by increasing the frequency of cell death.

In biological systems cell death may not be completely random but rather biased by age. Analytically, we showed that the age of the dead cell affects the expected sizes of cluster offspring. Here, we include a bias in the age at which cells die in the simulation by protecting reproducing cells from death; cells cannot die until a set amount of time has passed since their last reproduction. We expect this to act similarly to decreasing the death rate because fewer cells are susceptible to death. As such, we predict that the longer death is delayed the lower the final population. Instead, we find that delaying death has a variety of effects depending on the degree cap and the frequency of cell death (Figure 4D). The results match our expectations when the probability of death is low (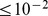) or clusters are not degree capped. In contrast, when the probability of death is high (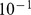), the number of cells in degree capped clusters increases if death is delayed. This effect is strongest when death is delayed only one round of cell reproduction, i.e. cells are susceptible when it has been at least one generation since their last reproductive event. As the delay gets longer the total number of cells decreases. For a degree cap of 4, delaying death for 5 rounds of cell reproduction still produces more cells than when there is no delay, but this is not true for a degree cap of 3. Thus, delaying death has different effects depending on the probability of death, the length of the delay, and the maximum node degree.

**Figure 5:**
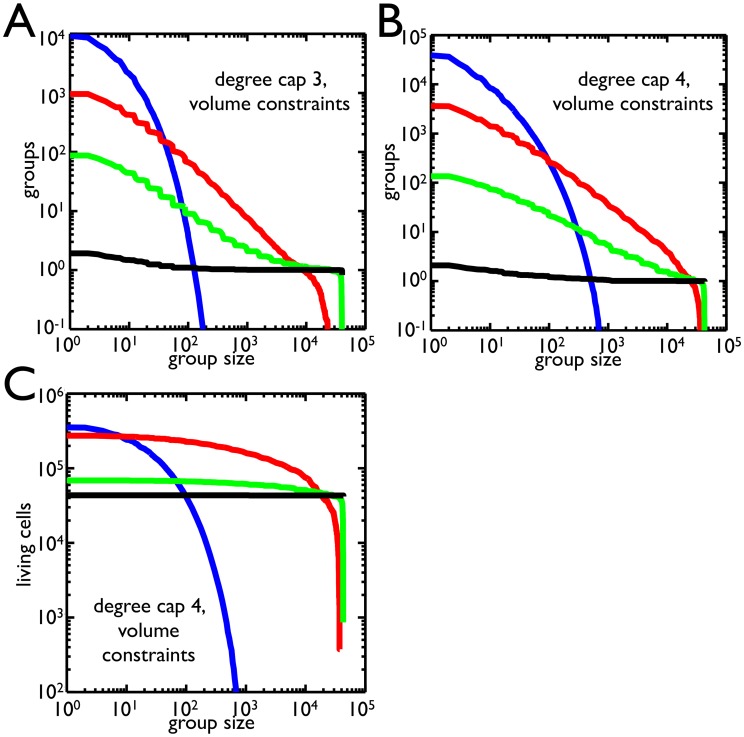
Selection for group size. **A**) The number of groups that satisfy size thresholds are shown for a degree cap of 3 for different probabilities of death: 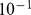 (blue), 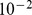 (red), 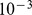 (green), 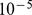 (black). As the group size increases, the number of groups above threshold drops. Small group size favors higher probabilities of death while large group size favors low probability of death. **B**) Same as A but with a degree cap of 4. The range in which 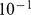 is dominant has expanded and 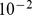 does better at group sizes above 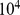. **C**) The number of cells within groups that satisfy size thresholds for a degree cap of 4 is shown for different probabilities of death (same color scheme). In contrast to B, the 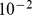 probability of death has a much larger range in which it is best. Comparing B and C, there is a region between 10 and 100 cells in which the 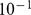 probability of death produces more groups but fewer cells in those groups than 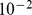.

Due to volume constraints and degree caps, apoptosis can increase both the number of cells and the number of clusters. Yet, the experimental regime rewarded cells that were in clusters above a certain size– this success might be measured as either the number of clusters or the number of cells in clusters. The frequency of cell death affects both the number and size distribution of clusters. To find which apoptosis rate yields the most clusters over different size thresholds, we compute the average number of clusters above threshold for different probabilities of death (Figure 5A for degree cap of 3 and 5B for degree cap of 4). For small cluster thresholds (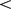
25 cells), the highest probability of death 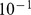 produces the most cluster offspring. As the cluster threshold increases to 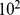 cells, the probability of death that leaves the most cluster offspring decreases to 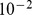. Larger size thresholds (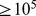) effectively reward clusters that never divide, and so the best strategy is to have the lowest probability of death (here, 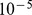). These trends also hold if the degree cap is 4, but the higher probabilities of death (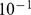 and 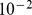) dominate for greater ranges of size thresholds. Moreover, these trends are the same if fitness is determined not by the number of clusters above threshold but rather the number of cells in those clusters. Once again, the higher probabilities of death are successful for size thresholds from 1 to 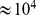. One notable difference is that for size thresholds between 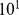 and 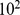, the highest probability of death, 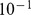, produces the most clusters but not the most cells– the 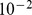 probability of death produces more cells in clusters above threshold.

**Figure 6:**
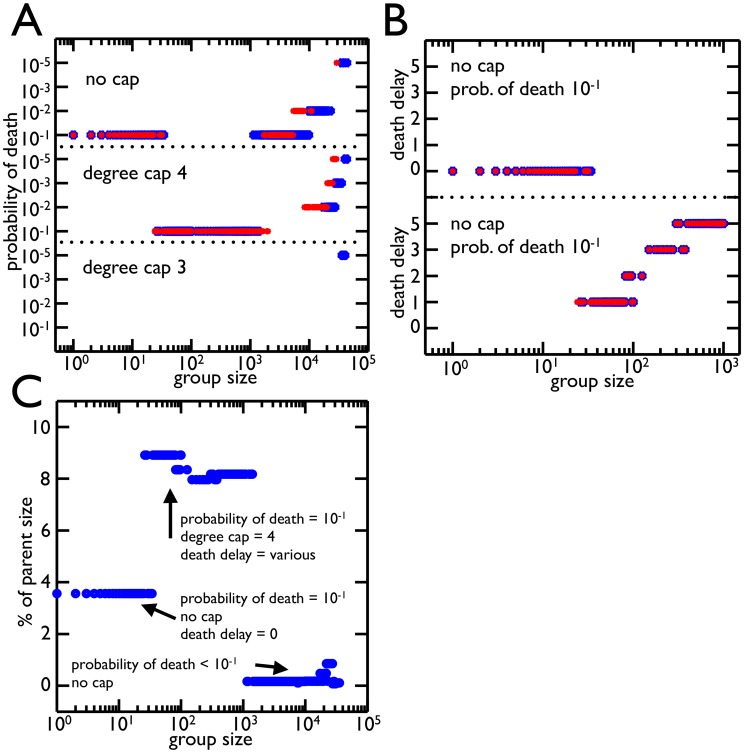
Best strategies for different group size selection. **A**) The probability of death that yields the most group offspring is shown for each threshold of group size for different rounds of cell reproduction (blue for 21, red for 19). The degree caps follow the same organization broken down by probability of death. In general the 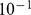 probability of death for a degree cap of 4 and no cap is the best strategy for most group sizes. Once the group size gets large (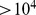) lower probabilities of death begin to win as it is advantageous not to divide large groups. **B**) The best strategies from A for group size thresholds ¡ 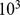 are distinguished by death delay (number of rounds without reproduction before a cell is susceptible to death). The optimal strategy of 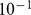 for no cap has no death delay, i.e. all cells are susceptible to death. For group size thresholds around 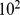 where degree cap of 4 with probability of death 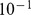 does better, the death delay increases with group size threshold. This increased death delay effectively lowers the probability of death. **C**) The average size of group offspring as a percent of parent size is shown for each optimal strategy from A (death delays included). The values are all under 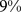 and are much smaller than those experimentally observed. There was, however, only one strategy which left more symmetrical groups (a degree cap of 3 with the highest probability of death).

In determining which apoptosis rate produces the most clusters, we assumed that the probability of death is an evolvable trait. The same may be true of other features related to cluster organization or cell death such as degree cap and the age bias of cell death (the death delay). To find which combination of these traits, “strategies”, yields the most clusters above threshold, we compare combinations of degree cap, probability of death, and death delay (Figure 6A, B). For each combination of traits we grow a population of clusters from a single cell, *Node 0*, and compute the distribution of cluster sizes. This is repeated 100 times and we compare the strategies across different cluster size thresholds. For weak thresholds that permit small clusters of less than 25 cells, the most clusters are left by those without degree caps who have a probability of death of 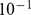 and no death delay. This strategy also produced the most living cells without considering cluster size thresholds (Figure 4D). For intermediate cluster thresholds between 25 and 1000 cells, a degree cap of 4 with a probability of death of 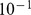 is best. As the size threshold increases within this range so does the optimal death delay. For cluster size selection between 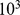 and 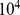 the best strategy shifts back to clusters without degree caps who have a probability of death of 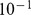 and death delays above 0. The largest cluster size selection (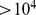) finds the lowest probabilities of death with all degree caps doing well. In general, these results hold if the number of generations in the simulations is reduced from 21 to 19.

We next consider how the best combinations of traits for different size thresholds fare in cluster offspring symmetry. We compute the average size of offspring cluster for the best strategies (Figure 6C) and find that they produce much smaller offspring than the 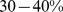 observed experimentally: 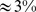 of the parent's size for small cluster selection, 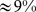 for intermediate clusters, and 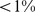 for large clusters. Although they fall short, only a degree cap of 3 with the highest probability of death left more symmetrical cluster offspring (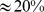). The symmetry of offspring did not compensate for the limits such a stringent degree cap places on population size.

**Figure 7:**
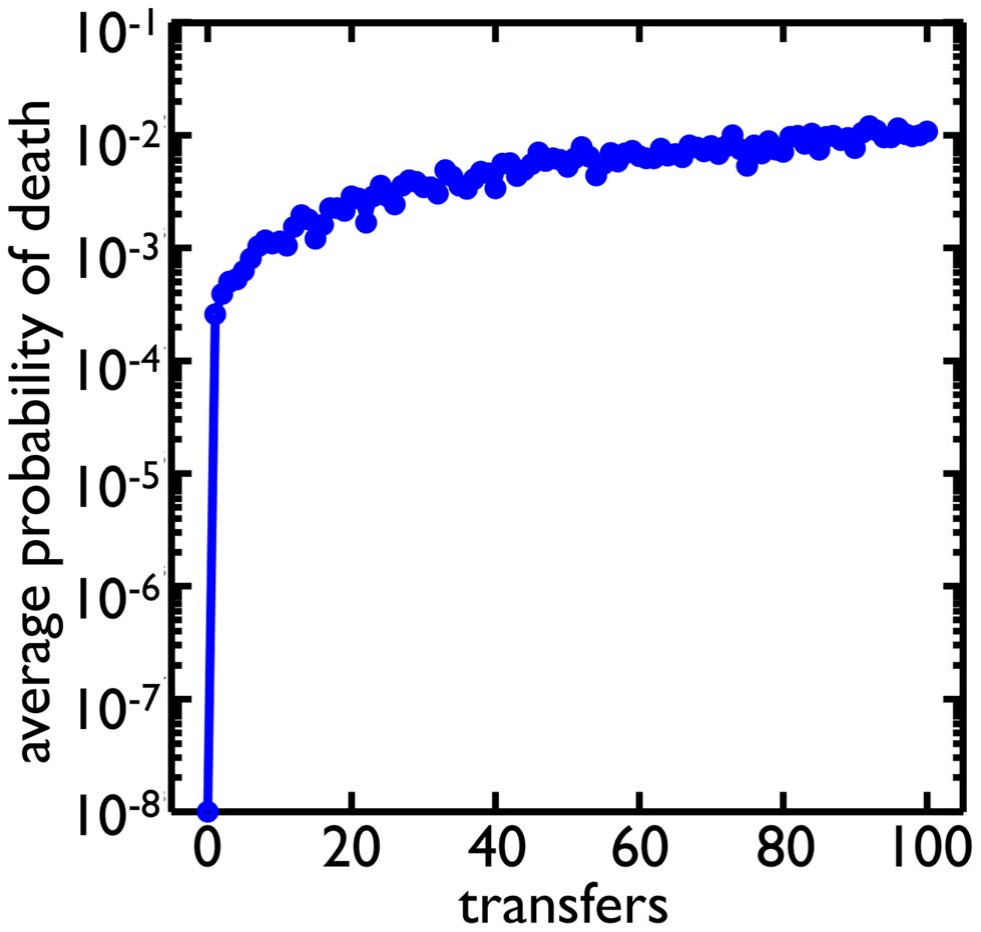
Evolution of higher rates of cell death. Clusters alternate between growth phases, in which the population increases by 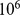 cells, and selective phases, in which clusters are randomly chosen based on size (see Methods) similar to the experimental regime. Cells can mutate and change their probability of death. Starting from a probability of death of 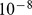, the average probability of death in the population evolves to around 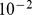.

To see if higher rates of cell death could be selected for in the context of evolving populations, we expand our simulations to include mutations and repeated cycles of growth and selection mimicking the transfers of the experiment [28]. We start the simulation with a single cell, *Node 0* with no degree cap and a low rate of cell death (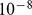). Along with each round of cell reproduction and death, there is a round of mutation. Cells mutate with a probability of 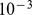 (assuming there are many mutations that affect the probability of cell death) and are assigned a new probability of death randomly sampled from a uniform distribution between 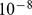 and 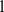. After the population grows 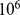 cell divisions, we randomly pick clusters based on their diameter as a proxy for settling speed until we have 10% of the population. This process of growth and selection is repeated for 100 transfers (Figure 7). The result is a rapid growth in the average probability of death for the population. The probability of death reaches 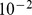 within a similar number of transfers as was found experimentally (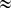
60 transfers [28]).

## Methods

Population simulations compute the growth and reproduction of clusters from a single cell, the first mutant to form clusters, *Node 0*. The simulation approach involves agent-based tracking of cell information, including the cluster it belongs to, the shell it is in, its parent cell, its number of offspring, the size of each of its branches (for biased link severance), and the time it last divided. Simulations have discrete time steps representing cell generations. At the start of a time step, all cells reproduce so long as they satisfy three conditions: 1. they are alive, 2. they have not reached the maximum degree (degree cap), and 3. there is room in the next shell where their offspring will reside (volume constraints). Following reproduction, we implement cell death. Initially all cells are susceptible and have a constant probability of death. This assumption is relaxed at times to delay death until a cell has been unable to divide a certain number of time steps, effectively protecting younger cells from death. If a cell dies then one of its links is randomly severed, biased by the number of cells along that branch (weighting). Dead cells remain attached to a cluster and no longer reproduce. Each simulation goes through 21 rounds of reproduction and death (cluster reproduction) which would allow planktonic cells to reach populations of 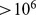 cells. At the end of the simulation, the distribution of cluster sizes is computed as well as the total number of living cells in those clusters. Simulations are done using the numerical software MATLAB (version 7.12.0.635 Natick, Massachusetts: The MathWorks Inc., 2011).

## Discussion

An experiment exploring the emergence of multicellularity observed the rapid evolution of groups from unicellular precursors in the yeast *Saccharomyces cerevisiae* when cultures were placed under selection for rapid settling through liquid medium [28]. Soon after the establishment of groups, cells also evolved a higher rate of apoptosis. Elevated cell death clearly lowers cell viability, but it would also seem to lower *group* viability. This is because settling selection favors large clusters and cell death facilitates group division, and thus size reduction. Why would natural selection favor elevated– as opposed to reduced– levels of apoptosis? Here we show that the organization of the group and the constraints imposed by its geometry are instrumental in understanding the functional consequences of apoptosis. By increasing the frequency of cell death, both the number of cells and groups can increase. Thus, a trait which is harmful to the cells that express it (they die) acts as a form of suicidal altruism and is beneficial to both the long-term number of cells and group entities once the group structure is considered. Furthermore, this trait may play a key role in the evolutionary transition to multicellularity.

With the transition from unicellularity to multicellularity there is an important shift in the level of organization and individuality [1]–[3]. A key requirement for multicellularity is formation of a cohesive group of cells. Group formation offers distinct advantages over a strictly solitary lifestyle such as protection from predation [31], [32], access to new niches [4], and survival in harsh environments [33]. However, for groups to qualify as units of selection, they must also possess the capacity to beget group offspring [27], [34], [35]. In this experimental yeast system, clusters grew in size and as a result of cell death or physical strain they fragmented and thereby reproduced. As a result, the yeast simultaneously evolved group formation and a mode of reproduction *de novo*. The later evolution of increased cell death led to more frequent cluster reproduction, thereby, linking reproductive self-sacrifice at the lower-level to fecundity at the higher-level. From a certain perspective, the fitness of the apoptotic lower-level units is subjugated to elevate the fitness of higher-level units, which is taken to be a hallmark of an evolutionary transition in individuality [1], [2], [27], [36], [37].

Interestingly, evolution of increased cell death also acts to stabilize the transition to multicellularity. If a cell with a higher rate of death were to leave the context of its collective, it would not fare well in competition with other cells who never formed groups (and never evolved greater apoptosis). In this way, the trait ratchets cells into a multicellular lifestyle by making them less competitive with their unicellular ancestors. This prevents abandonment of the collective and reversion to unicellularity. By tying the fate of cells to the fate of their groups, such context dependent traits stabilize primitive multicellular forms.

The amount of stabilization provided by a context dependent trait would likely depend on the fitness tradeoff between unicellular and multicellular life. More stabilization is expected from traits that severely hamper the fitness of cells outside the group context. It is unknown whether such stabilizing traits are common but with the yeast system analyzed in this paper there is robust selection for increased apoptosis rates. Rather than finding a narrow range of conditions that selected for moderately higher rates of cell death, we found strong selection for high rates of cells death (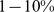) across a wide spectrum of cluster size thresholds. In fact, the only regime where increased cell death does not succeed is when groups need to be close to the maximum possible size. This regime selects for the lowest cell death rates and results in a single group encompassing the entire population. Otherwise, when size selection required minimum group sizes from 0 to 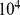, high rates of cell death allowed cells to circumvent limitations imposed by geometry. Interestingly, these limitations were of two different classes: limits to the number of connections and limits to space. The relative importance of each limitation depends on the geometry. Limits to connections are stronger for trees with a maximum node degree of 3 and under while limits to space are more restrictive when the maximum degree is 4 and higher. As a consequence, a gamut of different tree geometries encounter limitations to growth that robustly select for high rates of apoptosis.

Although using selective regimes based on Ratcliff *et. al*. experiment [28], favored high rates of cell death similar to those observed in the experiment, it did not match the same magnitude of offspring-parent ratio (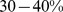). One reason for the mismatch with experimental data could be the compounding effects of cell death on the reproduction of the clusters. Experimental populations undergo repeated generations of cell reproduction and death which alters the geometric arrangements of cells. In comparison, the population simulations of Figures 4-7 contain nascent clusters who have grown from a single cell over the course of only 21 generations. They may not have had enough time to accumulate dead cells which alters their structure and promotes the birth of larger cluster offspring. Another possible reason for the mismatch could be due to our implementation of cell death. If cell death occurs under conditions of low nutrient concentration or build-up of cellular waste products, then cells in the center of clusters may be substantially more likely to die, which would produce relatively larger offspring clusters. Also, our models do not explicitly incorporate physical forces within growing clusters, which could affect likely break points, and thus, relative size of offspring clusters.

Our model implicitly assumes that the environment in which cells and groups grow is nutrient rich, and that the death of a cell provides the possibility for replacement by future cells. This allows apoptosis to overcome the cost of sacrificing a cell through the benefit of additional cellular reproduction. If, instead, the environment were nutrient poor and death of a cell did not guarantee replacement, then high rates of apoptosis would encounter an additional cost not reflected in our model, and would likely be less successful. It is possible that the model could be modified to consider cell survival as a function of crowdedness rather than cell fecundity. Cells in more crowded areas have less access to nutrients and by dying could create more access for neighbors, potentially improving their survival. These considerations lie outside the scope of this paper. In the experimental regime, as in the model, populations were grown in nutrient rich environments and so increased apoptosis led to both higher group and cell number. Still, it is important to recognize that the fitness consequence of traits depend on both the environment established by the group as well as its external environment.

While there have been many theoretical studies on the evolution of division of labor within multicellular organisms, modeling that division in the context of the multicellular geometry represents an under-explored direction. Considering the fitness implications of group geometry reveals that the group represents a novel, dynamic environment, one constructed bottom-up by individual cells. As such, variations in cellular physiology affect the geometry of the cluster, which in turn affects cell growth and survival. For example, if a cell has a morphology that only permits three connections to other cells, then the maximum possible cluster size will be much smaller than a cellular morphology that permits four connections. Similarly, different group formations impose different selective pressures on the cells within groups. The difference between three and four connections determines when cells will run out of space within the group. Although we considered a simple model with identical cells defined by just a few properties (maximum degree, apoptosis rate, and death delay), we found that these traits interact in surprising ways. For instance, increasing cell death increased the number of living cells but delaying death for cells– effectively reducing the apoptosis rate– had contrasting effects depending on the maximum degree and duration of the delay.

We only investigated how altering cell death affects cluster size and the number of cells/clusters in the population, but it is possible that cells could evolve different shapes, sizes, or behaviors which modify whole group-level traits. In fact, recent work has shown that strong selection for faster settling results in the evolution of larger, more elongate cells, which increase both group size and settling speed [38]. The environment faced by cells in clusters is not uniform: cells in the interior should experience a lower concentration of resources (as they must diffuse past other yeast that are consuming them) and higher concentrations of waste products. These environmental gradients provide reliable cues that could allow a cell to determine its position within the cluster. As cells change their location within the geometry and experience different internal environments, it may be advantageous to adopt different strategies or forms. This raises the possibility that selection can favor location-specific morphological or behavioral differentiation. Indeed, this may provide an evolutionary origin to primitive multicellular developmental programs.

## Supporting Information

Figure S1
**Optimal propagule sizes using the dynamic programming approach**. Here we programmed the recursion from Eq. 1 in SI and solved for the optimal division 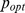 as a function of cluster size (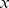) and time (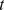). In our graphs we focus on a small range of sizes (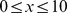) over five time steps. For all runs we assume 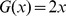 (clusters double every time step). In the program we set a maximum cluster size (of 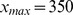; note, the largest a cluster could get within our focal range would be 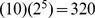). We vary the function 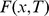 in these graphs and plot the optimal division size as the smaller fraction of a cluster after the split (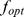). Note for 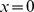, we set 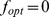 (although actually the optimal fraction is undefined) and for 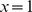, 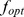 must be 0. Whenever distinct fractions give equivalent optimal strategies, the smallest fraction is plotted. **a**) Here we have a purely concave function 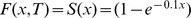, and we see that the optimal strategy is to split the cluster into two equal pieces. Of course, for clusters with an odd number of cells, this is impossible, but the optimal strategy is to divide the cluster as evenly as possible (e.g., a cluster of size 5 gets split into a cluster of size 3 and one of size 2, for 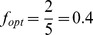). **b**) Suppose the survival function is purely concave (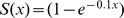 as before), but now maximal reproductive output is measured in terms of number of *cells* surviving selection, and not the number of clusters. Here, 
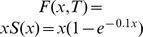
. In this case, it is optimal to avoid splitting under all conditions in our range. **c**) In cases where the survival function flips concavity across our range, optimal division can depend on size and time. Here 
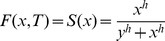
, where 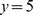 and 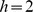. **d**) The same function is used here as in part (c), but 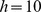.(TIF)

Text S1 **This document shows derivation of the dynamic programming approach and calculation of optimal division strategies.**
(PDF)
