# Supplementary figures and images for: Geometry Shapes Evolution of Early Multicellularity
Source: PLoS Comput Biol. 2014 Sep 18;10(9):e1003803. doi: 10.1371/journal.pcbi.1003803 (PMC4168977; doi:10.1371/journal.pcbi.1003803)

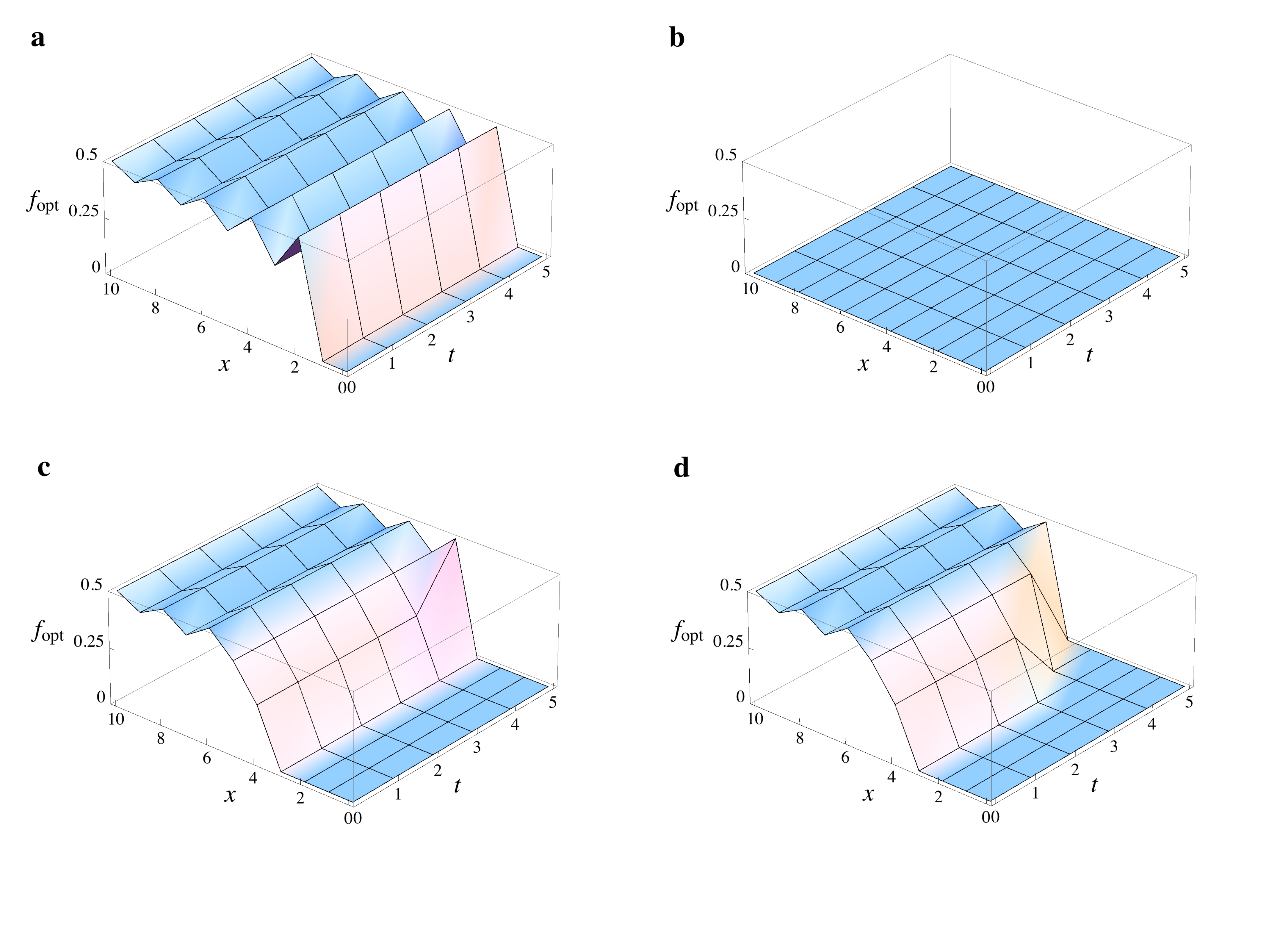

Supplement: Figure S1 — Optimal propagule sizes using the dynamic programming approach. Here we programmed the recursion from Eq. 1 in SI and solved for the optimal division as a function of cluster size () and time (). In our graphs we focus on a small range of sizes () over five time steps. For all runs we assume (clusters double every time step). In the program we set a maximum cluster size (of ; note, the largest a cluster could get within our focal range would be ). We vary the function in these graphs and plot the optimal division size as the smaller fraction of a cluster after the split (). Note for , we set (although actually the optimal fraction is undefined) and for , must be 0. Whenever distinct fractions give equivalent optimal strategies, the smallest fraction is plotted. a) Here we have a purely concave function , and we see that the optimal strategy is to split the cluster into two equal pieces. Of course, for clusters with an odd number of cells, this is impossible, but the optimal strategy is to divide the cluster as evenly as possible (e.g., a cluster of size 5 gets split into a cluster of size 3 and one of size 2, for ). b) Suppose the survival function is purely concave ( as before), but now maximal reproductive output is measured in terms of number of cells surviving selection, and not the number of clusters. Here, . In this case, it is optimal to avoid splitting under all conditions in our range. c) In cases where the survival function flips concavity across our range, optimal division can depend on size and time. Here , where and . d) The same function is used here as in part (c), but . (TIF) [file pcbi.1003803.s001.tif]
