# Supplementary material for: Geometry Shapes Evolution of Early Multicellularity
Source: PLoS Comput Biol. 2014 Sep 18;10(9):e1003803. doi: 10.1371/journal.pcbi.1003803 (PMC4168977; doi:10.1371/journal.pcbi.1003803)
Supplement: Text S1 — This document shows derivation of the dynamic programming approach and calculation of optimal division strategies. (PDF) [file pcbi.1003803.s002.pdf]

Supplement: Dynamic Programming Results

In our model, clusters grow over T time steps and gravity selection then occurs. At the beginning of each time step, cluster division can occur. Following division, clusters grow, such that any cluster of size x cells will be $G(x)$ cells by the end of the time step. Time point $t = 0$ marks the beginning of the first time step and time $t = T$ marks the point of gravity selection. Upon selection, a cluster of x cells survives with probability $S(x)$, which we take to be a non-decreasing function. The maximal reproductive output for a cluster of size x at time t is given by $F(x, t)$. For us, this output function is simply a means to determine the optimal way for clusters to split, which can depend on both size and time. In our scheme, a cluster of x cells can split into two clusters of sizes p and $x - p$ (where $0 \leq p \leq x/2$). Because it is possible for the cluster not to split (i.e., if $p = 0$), we can simultaneously address the optimal rate of division along with optimal (a)symmetry.

A backwards recursion for maximal reproductive output can be formulated:

$$F(x,t) = \max_{0 \leq p \leq x/2} (F(G(p), t+1) + F(G(x-p), t+1)) \tag{1}$$

Suppose that $G(x) = ax$ (where a is an integer greater than unity) and $F(x, t + 1)$ is concave. We note that $F(x, t+1)$ is only defined for integer values of x, so the standard requirement ($\frac{d^2F(x,t+1)}{dx^2} \leq 0$) is replaced by the following condition:

$$(F(x+1,t+1) - F(x,t+1)) - (F(x,t+1) - F(x-1,t+1)) \leq 0 \tag{2}$$

If condition 2 holds for all values of x, then we have the following proposition.

Proposition

Given that $F(x, t + 1)$ is concave by condition 2; for all integer values of p, where $0 \leq p \leq \frac{x}{2}$:

$$F(ap, t+1) + F(a(x-p), t+1) \leq \begin{cases} 2F\left(\frac{ax}{2}, t+1\right) & \text{if } x \text{ is even} \\ F\left(\frac{a(x+1)}{2}, t+1\right) + F\left(\frac{a(x-1)}{2}, t+1\right) & \text{if } x \text{ is odd} \end{cases} \tag{3}$$

Proof

Here we use a proof by induction. Consider the case where x is even. Let $n = a\left(\frac{x}{2} - p\right)$ (for any defined value of p, n will be some non-negative integer value). Condition 3 can be rewritten as:

$$F\left(\frac{ax}{2}, t+1\right) \geq \frac{F\left(\frac{ax}{2} - n, t+1\right) + F\left(\frac{ax}{2} + n, t+1\right)}{2} \tag{4}$$

Here, we will consider all non-negative integer values of n (even those that don't correspond to an integer value of p). Condition 4 clearly holds for $n = 0$. Additionally, it holds for $n = 1$ because condition 2 can be rewritten (with x replaced by $\frac{ax}{2}$) as

$$F\left(\frac{ax}{2}, t+1\right) \geq \frac{F\left(\frac{ax}{2} - 1, t+1\right) + F\left(\frac{ax}{2} + 1, t+1\right)}{2} \tag{5}$$

We now assume that condition 4 holds for n and show that it must hold for $n + 1$. If $F(x, t + 1)$ is a concave function, then we are guaranteed

$$F(x-1,t+1) \leq 2F(x,t+1) - F(x+1,t+1) \tag{6}$$

$$F(x+1,t+1) \leq 2F(x,t+1) - F(x-1,t+1) \tag{7}$$

Using conditions 6 and 7, the following holds:

(8)
$$\frac{F\left(\frac{ax}{2}+(n+1),t+1\right)+F\left(\frac{ax}{2}-(n+1),t+1\right)}{2} \leq F\left(\frac{ax}{2}+n,t+1\right)+F\left(\frac{ax}{2}-n,t+1\right) - \frac{F\left(\frac{ax}{2}+(n-1),t+1\right)+F\left(\frac{ax}{2}-(n-1),t+1\right)}{2}$$

The following condition holds

$$\frac{F\left(\frac{ax}{2}+n,t+1\right)+F\left(\frac{ax}{2}-n,t+1\right)}{2} - \frac{F\left(\frac{ax}{2}+(n-1),t+1\right)+F\left(\frac{ax}{2}-(n-1),t+1\right)}{2} \leq 0 \quad (9)$$

To show condition 9, we note that condition 7 ensures

(10)
$$F\left(\frac{ax}{2}+n,t+1\right)-F\left(\frac{ax}{2}+(n-1),t+1\right)+F\left(\frac{ax}{2}-n,t+1\right)-F\left(\frac{ax}{2}-(n-1),t+1\right) \leq F\left(\frac{ax}{2}+(n-1),t+1\right)-F\left(\frac{ax}{2}+(n-2),t+1\right)+F\left(\frac{ax}{2}-n,t+1\right)-F\left(\frac{ax}{2}-(n-1),t+1\right)$$

However condition 7 also ensures

(11)
$$F\left(\frac{ax}{2}+(n-1),t+1\right)-F\left(\frac{ax}{2}+(n-2),t+1\right)+F\left(\frac{ax}{2}-n,t+1\right)-F\left(\frac{ax}{2}-(n-1),t+1\right) \leq F\left(\frac{ax}{2}+(n-2),t+1\right)-F\left(\frac{ax}{2}+(n-3),t+1\right)+F\left(\frac{ax}{2}-n,t+1\right)-F\left(\frac{ax}{2}-(n-1),t+1\right)$$

And the same substitution can be repeatedly applied, which yields

(12)
$$F\left(\frac{ax}{2}+n,t+1\right)-F\left(\frac{ax}{2}+(n-1),t+1\right)+F\left(\frac{ax}{2}-n,t+1\right)-F\left(\frac{ax}{2}-(n-1),t+1\right) \leq F\left(\frac{ax}{2}-(n-1),t+1\right)-F\left(\frac{ax}{2}-n,t+1\right)+F\left(\frac{ax}{2}-n,t+1\right)-F\left(\frac{ax}{2}-(n-1),t+1\right)=0$$

Thus, condition 9 follows.

Condition 9 shows that condition 8 can be rewritten as

$$\frac{F\left(\frac{ax}{2}+(n+1),t+1\right)+F\left(\frac{ax}{2}-(n+1),t+1\right)}{2} \leq \frac{F\left(\frac{ax}{2}+n,t+1\right)+F\left(\frac{ax}{2}-n,t+1\right)}{2} \quad (13)$$

Given that we are assuming that condition 4 holds for n, it now follows

$$\frac{F\left(\frac{ax}{2}+(n+1),t+1\right)+F\left(\frac{ax}{2}-(n+1),t+1\right)}{2} \leq F\left(\frac{ax}{2},t+1\right) \quad (14)$$

Thus, condition 4 holds for $n+1$. Thus, this condition will hold for all integer values of n, which certainly ensures that it will hold for all integer values of p (where $0 \leq p \leq \frac{x}{2}$). The case where x is odd follows a similar argument. This completes the proof. ■

Condition 4 is essentially an instance of Jensen's inequality. Using Eq. 1 and condition 3,

$$F(x,t)=\begin{cases} 2F\left(\frac{ax}{2},t+1\right) & \text{if } x \text{ is even} \\ F\left(\frac{a(x+1)}{2},t+1\right)+F\left(\frac{a(x-1)}{2},t+1\right) & \text{if } x \text{ is odd}\end{cases} \quad (15)$$

Suppose that x is even; then Eq. 15 ensures

(16)
$$\begin{aligned}(F(x,t)-F(x-1,t))-(F(x-1,t)-F(x-2,t)) &= 2F\left(\frac{ax}{2},t+1\right)-2F\left(\frac{ax}{2},t+1\right) \\ &\quad -2F\left(\frac{a(x-2)}{2},t+1\right)+2F\left(\frac{a(x-2)}{2},t+1\right) \\ &= 0\end{aligned}$$

Suppose that x is odd; then Eq. 15 ensures

(17)

$$\begin{aligned}(F(x,t)-F(x-1,t))-(F(x-1,t)-F(x-2,t)) &= F\left(\frac{a(x+1)}{2},t+1\right)+F\left(\frac{a(x-1)}{2},t+1\right)\\ &-2F\left(\frac{a(x-1)}{2},t+1\right)-2F\left(\frac{a(x-1)}{2},t+1\right)\\ &+F\left(\frac{a(x-1)}{2},t+1\right)+F\left(\frac{a(x-3)}{2},t+1\right)\\ &=\left[F\left(\frac{a(x+1)}{2},t+1\right)-F\left(\frac{a(x-1)}{2},t+1\right)\right]\\ &-\left[F\left(\frac{a(x-1)}{2},t+1\right)-F\left(\frac{a(x-3)}{2},t+1\right)\right]\end{aligned}$$

Because $F(x,t+1)$ is concave by assumption, this means that the right-hand side of Eq. 17 is less than or equal to zero; thus, for all relevant x

$$(F(x,t)-F(x-1,t))-(F(x-1,t)-F(x-2,t))\leq 0 \tag{18}$$

This means that if $F(x,t+1)$ is concave over integer values of x, then $F(x,t)$ will be as well. Thus, if $F(x,T)=S(x)$ is concave, then $F(x,t)$ will be concave for all values of t. This means that there will be no better strategy than splitting the group into two equal pieces (or as close as possible).

If it were possible for $F(x,t+1)$ to be convex for all integer values $((F(x+1,t+1)-F(x,t+1))-(F(x,t+1)-F(x-1,t+1))\geq 0)$, then a similar argument would show that $F(x,t)$ will be convex as well. In such a case, it would be best for the cluster not to split at all ($p=0$ is the optimal value). Given that $S(x)$ is a probability (and thus bounded at unity) this function cannot be convex for all values of x. Therefore, if $F(x,T)=S(x)$, then it will generally not be the case that $F(x,t)$ is convex for arbitrary values of x and t. It is possible for $S(x)$ (and $F(x,t)$) to be convex for some values of x and concave for other values. In such a case, it is possible for the optimal division strategy to depend on size and time. For instance, in Figure S1, the results of a program to calculate the optimal division values (p as a function of x and t) using Eq. 1 are given for a set of different functions for $F(x,T)$ (in the figure, we do impose a maximum size for a cluster). Thus, we see that there are circumstances where a cluster should not divide at certain sizes and divide evenly at other sizes.
